# Predictive Constitutive Modelling of Oxidation-Induced Degradation in 2.5D Woven C/SiC Composites

**DOI:** 10.3390/ma19020307

**Published:** 2026-01-12

**Authors:** Tao Wu, Yukang Wang, Wenxuan Qi, Xingling Luo, Peng Luo, Xiguang Gao, Yingdong Song

**Affiliations:** 1 Jiangsu Province Key Laboratory of Aerospace Power System, College of Energy and Power Engineering, Nanjing University of Aeronautics and Astronautics, Nanjing 210016, China; taowu017@nuaa.edu.cn (T.W.); sz2402054@nuaa.edu.cn (Y.W.); luopeng@nuaa.edu.cn (P.L.); gaoxiguang@nuaa.edu.cn (X.G.); ydsong@nuaa.edu.cn (Y.S.); 2 Key Laboratory of Fundamental Science for National Defense-Advanced Design Technology of Flight Vehicle, Nanjing University of Aeronautics and Astronautics, Nanjing 210016, China; 3 Key Laboratory of Aero-Engine Thermal Environment and Structure, Ministry of Industry and Information Technology, Nanjing 210016, China; 4 State Key Laboratory of Mechanics and Control Mechanical Structures, Nanjing University of Aeronautics and Astronautics, Nanjing 210016, China

**Keywords:** C/SiC composites, oxidative damage, constitutive model, high-temperature mechanical properties, RVE model

## Abstract

Oxidation can lead to intrinsic degradation and loss in the load-bearing capacity of ceramic matrix composites (CMCs) in high-temperature service, thereby compromising structural integrity and operational safety. To elucidate the mechanism of its oxidation effects, this study predicted the oxygen diffusion coefficient within 2.5D woven C/SiC fibre bundles based on gas diffusion and oxidation kinetics theory, and subsequently constructed a meso-scale constitutive model incorporating oxidation damage and fibre defect distribution. Furthermore, a micro-scale framework for yarns was established by integrating interfacial slip behaviour, and an RVE model for 2.5D woven C/SiC was constructed based on X-ray computed tomography reconstruction of the actual microstructure. Building upon this foundation, an oxidation constitutive model applicable to loading–unloading cycles was proposed and validated through high-temperature oxidation tests at 700 °C, 900 °C, and 1100 °C. Results demonstrate that this model effectively characterizes the strength degradation and stiffness reduction caused by oxidation, enabling prediction of CMCs’ mechanical properties under oxidizing conditions and providing a physics-based foundation for the reliable design and life assessment of C/SiC components operating in oxidizing environments.

## 1. Introduction

Ceramic matrix composites (CMCs) are widely used in aero-engine hot-end components and hypersonic vehicle thermal protection systems due to their high-temperature stability, corrosion resistance and lightweight and high-specific-strength characteristics [1,2,3]. Among them, C/SiC composites have stability advantages in complex thermal environments, but are prone to significant oxidation processes under oxygen-rich high-temperature service conditions. The pores, microcracks and fibre–matrix interfaces within the material provide channels for oxygen diffusion, making the gradual evolution of oxidation damage a key factor limiting its lifetime and structural reliability [4,5,6].

Existing studies show that the oxidation behaviour of C/SiC exhibits typical staged characteristics and is controlled by the diffusion mechanism together with the interfacial structure [7,8,9]. In the lower temperature range (500–800 °C), oxygen mainly diffuses along the initial defects, and the material is dominated by the accumulation of mass loss. As the temperature rises (800–1100 °C), the permeability of the fibre bundles is enhanced and the thermally expanding microcracks are gradually connected; the gas transport mechanism is dominated by molecular diffusion and Knudsen diffusion, and interfacial depletion occurs in tandem with the weakening of the fibres [10]. At higher temperatures (above 1100 °C), the SiC matrix generates oxide films [11], the oxidation behaviour changes from reaction-controlled to film growth-controlled, its densification determines the ability of further oxygen diffusion, and the material is prone to enter into rapid embrittlement when the integrity of the film structure cannot be maintained [12,13,14]. The above multi-stage, multi-mechanism synergistic effect makes C/SiC in an oxidation environment present significant non-uniformity and multi-scale evolution characteristics.

Regarding the degradation of properties under oxidation conditions, studies have been conducted to analyze the damage modes, interfacial behaviours and fracture characteristics of C/SiC composites through macroscopic mechanical tests and microscopic characterization [15,16,17]. Vagaggini et al. [18] proposed a theoretical framework based on hysteresis response analysis, which was used to identify the mechanical characterization of different damage modes, such as debonding of fibre/matrix interfaces, cracking of the matrix, and damage of the fibres, under cyclic loading. Zhang et al. [19] studied the oxidation service behaviour of C/SiC by loading and fracture characterization in high-temperature environments, focusing on the change rule of the material’s mechanical response and fracture characteristics under the action of an oxidizing medium. Cheng et al. [20] carried out experimental and analytical studies around the high-temperature fracture mechanism of two-dimensional C/SiC, discussing the evolution trend of crack extension paths and interfacial behaviour under the action of temperature. Sullivan et al. [21] regarded C/SiC as a continuous medium and constructed a numerical model of coupled oxidation kinetics and gas diffusion, which was used to analyze the evolution of the oxide layer and diffusion channels and the influence of the pore and interface states on the oxidation process. However, existing models predominantly rely on empirical strength reduction or parameter fitting approaches, making it challenging to establish mechanical characterization methods consistent with oxidation mechanisms. This is particularly true for woven C/SiC composites exhibiting spatial connectivity and anisotropic characteristics, for which no suitable mechanical characterization framework currently exists to assess oxidation damage [22,23,24]. This limitation constrains the ability to predict the performance and design the structure of C/SiC composites under high-temperature service conditions.

Based on the above problems, this paper proposes an intrinsic model for predicting the mechanical behaviour of 2.5D woven C/SiC composites [25] (i.e., a multilayer woven architecture in which warp/weft yarns form stacked in-plane layers and a portion of binder warp yarns periodically interlock adjacent layers to provide limited through-thickness reinforcement, intermediate between 2D laminates and fully 3D woven composites) in a high-temperature oxidation environment. The model establishes a yarn ontology model by studying the microscopic oxidation behaviour at the fibre interface and matrix levels through X-ray computed tomography (X-CT) imaging. The representative volume element (RVE) of the material is constructed by coupling the microscopic oxidation damage with the macroscopic mechanical response through a multiscale analysis method. A computationally efficient and versatile oxidation constitutive model has been further developed to predict the mechanical properties of materials under high-temperature oxidation environments.

## 2. Materials and Methods

### 2.1. Material Preparation

**Figure 1:**
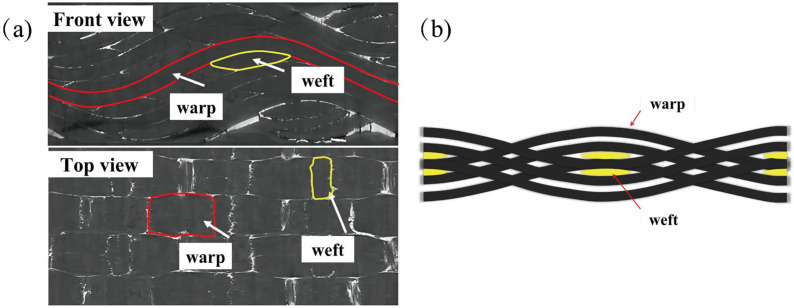
Representation of 2.5D woven C/SiC composites’ preform structure. (**a**) Photograph of the specimen; (**b**) schematic diagram.

The 2.5D woven C/SiC composites were made by the Beijing Institute of Aeronautical Materials (Beijing, China) at 1200 °C using chemical vapour infiltration (CVI) technology. The preform was made from 5 k (T-300) carbon fibre bundles in a 2.5D shallow-curved interlocked structure. First, a 0.2 µm layer of pyrolytic carbon was deposited onto the fibre surface using a process called chemical vapour deposition (CVD). Second, the β-SiC matrix was introduced using CVI. The resulting composite material was 3 mm thick. It had a density of 2.17 g cm^−3^, with about 32% of its volume made up of fibres, and a porosity of 10%. The structure of the form is shown in Figure 1. This preform is formed by interlacing warp and weft yarns, with the warp and weft yarns arranged at right angles to each other to create a tightly woven structure.

### 2.2. Oxidation Exposure and Tensile Loading–Unloading Tests

**Figure 2:**
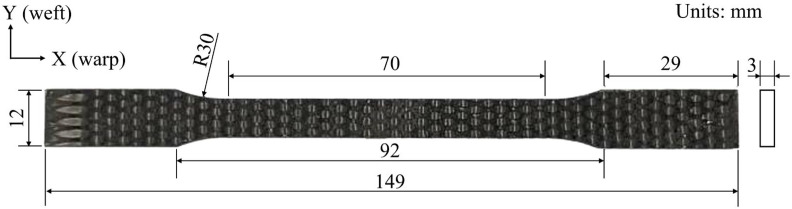
Dimensions of tensile test specimen.

**Figure 3:**
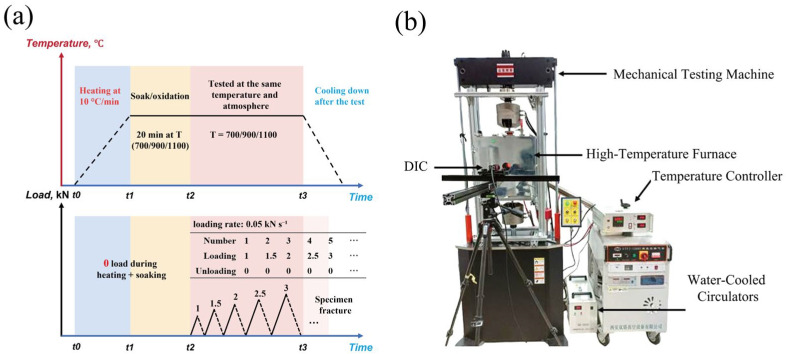
Oxidation exposure and tensile loading–unloading test protocol. (**a**) Schematic temperature–time and load–time histories; (**b**) high-temperature mechanical test system.

In this study, all 2.5D woven C/SiC composite specimens were subjected to a grinding and polishing process. Dog-bone-shaped samples with the geometry illustrated in Figure 2 were prepared by means of waterjet cutting. High-temperature oxidation loading and unloading tests were carried out on an INSTRON Model 8502 (INSTRON System Corp., Norwood, MA, USA) equipped with a high-temperature silicon molybdenum furnace GSL-1600 (1600 °C, ±5 °C) and a water-cooled fixture of our own design. The specimens were first heated in laboratory air to the target temperature (700 °C, 900 °C, or 1100 °C) at a heating rate of 10 °C/min. During heating, the applied load was controlled and maintained at approximately 0 N to avoid mechanical loading in the heating stage. After reaching the target temperature, the specimens were held (soaked) for 20 min to obtain the prescribed oxidation exposure. Immediately after the soaking period, tensile loading–unloading tests were performed without cooling. A room-temperature condition of 25 °C was used as the control. All tests were performed under force control with a load rate of 0.05 kN s^−1^ until final fracture. The cyclic loading–unloading measurements followed the prescribed stepwise protocol, where each cycle consisted of loading to a predefined peak force and immediately unloading back to ∼0 N before the next cycle. Force and crosshead displacement were recorded by the testing machine, and the axial strain within the gauge section was measured synchronously by a 2D digital image correlation (2D-DIC) system using prefabricated high-temperature-resistant speckle patterns [26]. Testing-machine signals were acquired at a sampling rate of 500 Hz. The temperature–time and load–time histories of the protocol are summarized in Figure 3a, and the high-temperature testing system is shown in Figure 3b.

### 2.3. Microstructural Characterization

The microstructure and fracture damage morphology of the composite material were characterized using scanning electron microscopy (SEM-Gemini 300, Zeiss, Oberkochen, Germany). Due to the poor electrical conductivity of the C/SiC composite, it was gold-sputtered prior to imaging.

## 3. Test Results and Discussions

### 3.1. Microstructure and Damage Morphology of 2.5D Woven C/SiC Composites

**Figure 4:**
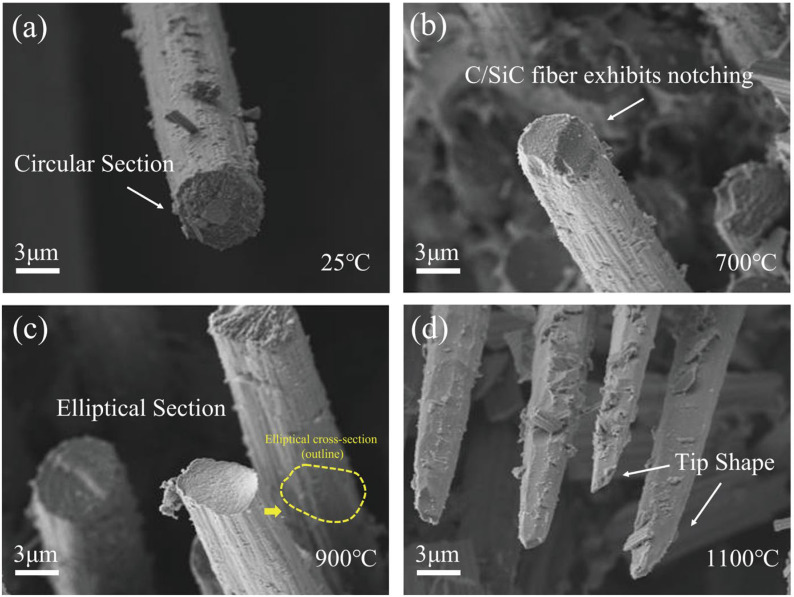
Fracture morphology of characteristic fibres at different temperatures. (**a**) Unoxidized fibres; (**b**) oxidized environment at 700 °C; (**c**) oxidized environment at 900 °C; (**d**) oxidized environment at 1100 °C.

The fracture morphology at the fibre ends before and after tensile loading is illustrated in Figure 4, providing insight into the damage evolution of the C/SiC composites. As shown in Figure 4a, the room-temperature unoxidized fibre fracture cross-section is a complete circle. The oxidation behaviour of carbon fibres changes significantly with increasing oxidation temperature. In Figure 4b, the fibre surface begins to erode under oxidizing conditions at 700 °C, with the formation of distinctive “notches’’ in localized areas. These notches appear mainly near the fibre–matrix interface and form deep depressions on the fibre surface. This is closely related to the localized oxidation of the fibres in the cracked areas, where the inhomogeneity of the oxidation not only weakens the load-bearing capacity of the fibres, but also induces stress concentration problems. According to Zhang et al. [27], the fibres in ceramic matrix composites exhibit significant inhomogeneity after oxidation, and usually notches are formed in the more severely oxidized areas, leading to a reduction in the effective cross-sectional area and an intensification of the local stress concentration problem. In addition, the tensile test results of Yang et al. [28] also showed that the failure of carbon fibre-reinforced composites under oxidized conditions usually starts from these notched parts formed due to oxidation. When the number of notches increases and the depth deepens, the overall mechanical properties of the material decrease significantly, which becomes the main controlling factor for the failure of composites.

As shown in Figure 4c,d, in an oxidation environment at 900 °C, the fibre fracture erosion phenomenon is more obvious, and its cross-sectional morphology gradually evolves from a round shape in the unoxidized state to an elliptical shape. This reduction in cross-sectional area directly weakens the load-bearing capacity of the fibre and reduces the overall strength of the fibre. The cross-sectional morphology of the fibres further evolves to a tip shape in the oxidized environment at 1100 °C. This change suggests that the edge portion of the fibre material is not as strong as it was in the unoxidized state. This change indicates that the edge of the fibre material oxidizes significantly faster than the centre, causing the fibres to shrink along the periphery towards the centre. The tip shape is more likely to lead to stress concentration, which further increases the possibility of fibre fracture.

**Figure 5:**
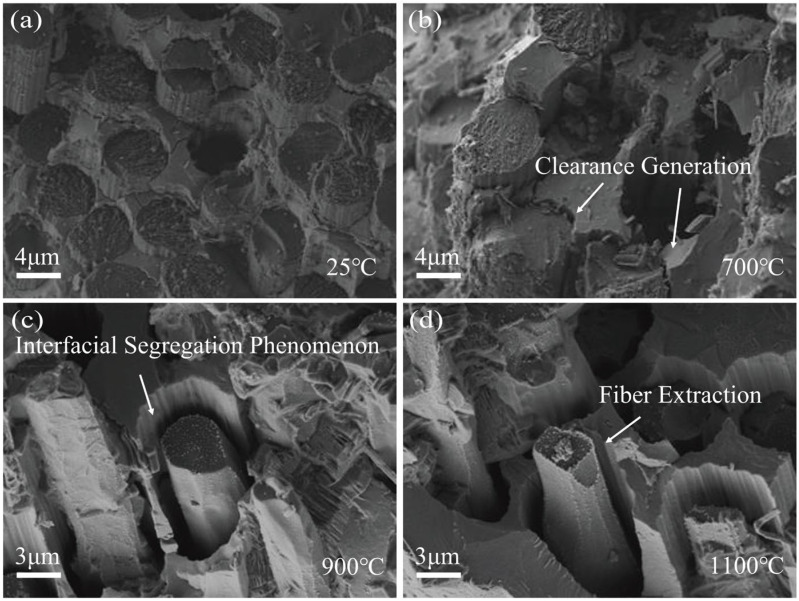
Oxidation processes at the interface. (**a**) Unoxidized fibres; (**b**) oxidized environment at 700 °C; (**c**) oxidized environment at 900 °C; (**d**) oxidized environment at 1100 °C.

The microstructural evolution within the three-component bonding region during tensile loading and unloading under high-temperature oxidation is depicted in Figure 5. As shown in Figure 5a, the debonded fibre/matrix interface undergoes abrasion and produces smaller gaps, but most of the interfacial layers are still wrapped around the surface of the fibres, which leads to a good load transfer effect. In the high-temperature oxidation environment, the emergence of cracks and their subsequent expansion not only significantly weaken the overall bearing capacity of the material, but also provide a diffusion path for the penetration of oxidizing gasses, thus accelerating the degradation process of the fibre/matrix interface. In Figure 5c,d, the obvious interfacial debonding phenomenon between the fibres and the matrix, accompanied by fibre pull-out behaviour, indicates that the interfacial bond strength has been significantly reduced. Due to the selective oxidation reaction of the interfacial phase, the interfacial region around the fibre also has obvious pores and microscopic damage.

### 3.2. Stress–Strain Curves of 2.5D Woven C/SiC Composites in Oxidative Loading/Unloading Tensile Tests

**Figure 6:**
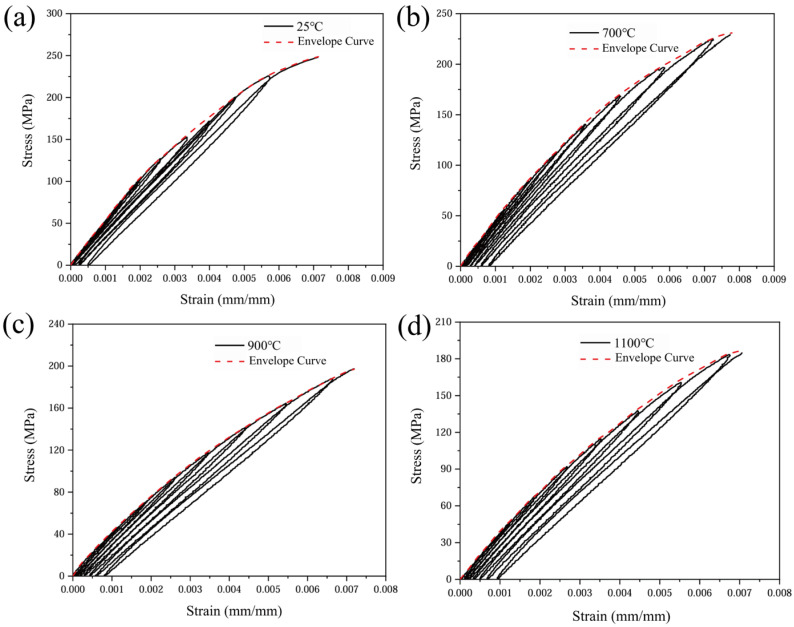
Stress–strain curves of 2.5D woven C/SiC composites under loading and unloading at different temperatures. (**a**) 25 °C; (**b**) 700 °C; (**c**) 900 °C; (**d**) 1100 °C.

The tensile loading–unloading stress–strain responses at various oxidation temperatures, together with the corresponding upper envelope curves [29], are presented in Figure 6. By comparing the stress–strain test data, it was found that both the maximum strength and the strain to failure of the CMC materials decreased gradually with increasing temperature. Specifically, the maximum tensile strength of the material decreases from 263.63 MPa at 25 °C to 183.75 MPa at 1100 °C, and the strain to failure gradually decreases from 7.88 × 10^−3^ to 7.3 × 10^−4^. When the material undergoes tensile loading and unloading, the degree of damage increases significantly with increasing oxidation temperature. Since the damage accumulated prior to unloading degrades the load-carrying capacity, once reloading exceeds the previously attained maximum stress, the stress–strain trajectory deviates from the original upper envelope curve and exhibits pronounced softening; this phenomenon is particularly prominent in the subsequent nonlinear phase. The root cause of this phenomenon is the continuous weakening of the microstructure by high-temperature oxidation: the cross-sectional area of the C-fibre bundles, which are the main load-carrying phase, is reduced and their strength is degraded by oxidation, leading to the loss of matrix–fibre synergy and a macroscopic decrease in stiffness and strength.

The upper envelope curves indicate an approximately linear elastic response at low stress levels, suggesting that the yarn architecture and mechanical properties remain essentially unchanged in this regime. When the load exceeds the critical value, the brittle matrix cracks sequentially, and the crack density increases with the elevated stress and induces interfacial debonding and slip. Damage accumulation, interfacial friction and interlayer extrusion together lead to decreasing cutline stiffness, and the stress–strain response becomes nonlinear [30]. Temperature elevation causes premature termination of the linear segment and a significant reduction in the nonlinear threshold. Under oxidizing conditions, inelastic deformation becomes more pronounced: the slope and curvature of the nonlinear segment increase with temperature, and cyclic loading exhibits distinct hysteresis loops, the area of which expands with rising temperature. This quantitatively confirms heightened energy dissipation and reveals that damage evolution rates accelerate significantly under high-temperature oxidation coupling.

**Figure 7:**
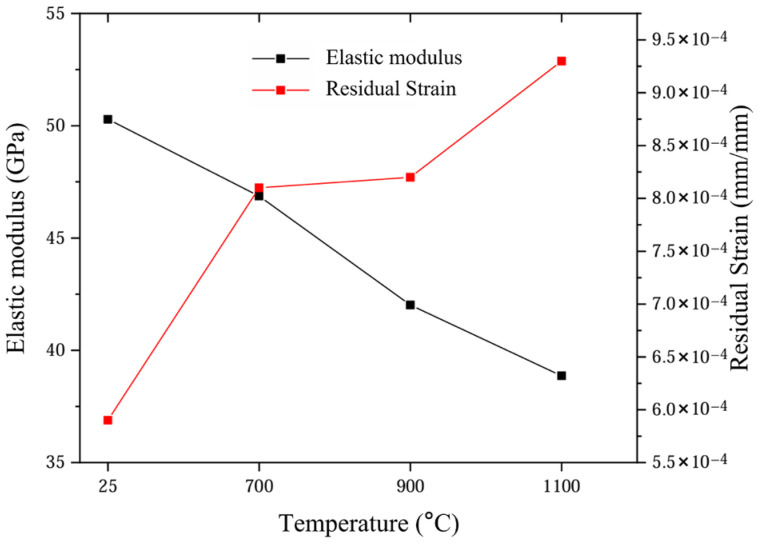
Variation in mechanical properties of 2.5D woven C/SiC composites.

The variation in the initial linear modulus with oxidation temperature is summarized in Figure 7, showing a monotonic decrease from 50.29 MPa at 25 °C to 38.86 MPa at 1100 °C (approximately 23%). After the loading–unloading cycle, the strain could not return to zero, and the residual strain increased linearly with temperature (5.9 × 10^−4^ → 9.3 × 10^−4^), which quantitatively confirmed that the irreversible damage induced by high-temperature oxidation continued to accumulate.

## 4. Constitutive Model Incorporating Oxidation Effect

**Figure 8:**
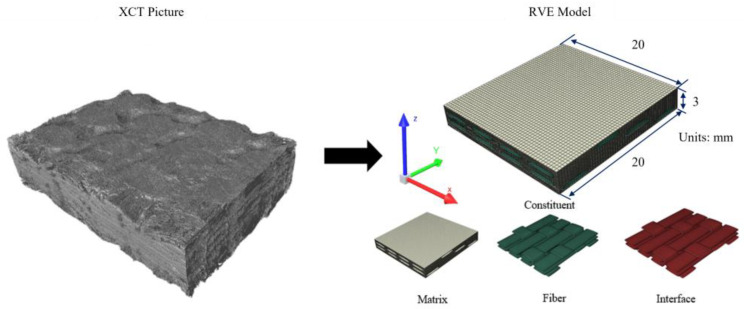
Weaving RVE microscopic structures.

Woven ceramic matrix composites consist of periodically arranged yarn reinforcements, which are woven in specific patterns to bear primary loads and whose properties determine the material’s macroscopic mechanical behaviour. This study adopts a “yarn-to-bulk’’ approach: First, a mesoscale yarn oxidation constitutive model is established, coupling gas diffusion with component oxidation mechanisms to characterize the mechanical response of individual yarns under oxidizing conditions. Subsequently, a 2.5D woven structure model is constructed using the RVE (representative volume element) method, embedding the yarn constitutive model to obtain macroscopically equivalent mechanical properties. This work employs X-CT to scan 2.5D woven C/SiC composites, followed by three-dimensional geometric modelling of their RVE using TexGen (v3.13.0) software [31], as shown in Figure 8.

### 4.1. Oxidation Kinetics Model

In porous media, the diffusion mechanism of gases varies with the ratio of pore size to the mean free path of gas molecules. Oxygen diffusion within cracks of ceramic matrix composites exhibits mixed diffusion characteristics, as the gas mean free path falls within the range of 0.1 to 100 times the molecular free path. Consequently, the diffusion process is described using equivalent diffusion, with the coefficient expressed as [32]:

(1) $${\bar{D}}^{-1}=D_{F}^{-1}+D_{K}^{-1},$$

where *D_F_* and *D_K_* represent the Fick diffusion coefficient and Knudsen diffusion coefficient, respectively. The definitions of all symbols are provided in Appendix A, Table A2. *D_F_* and *D_K_* can also be expressed as:

(2) $$\left\{\begin{array}{c} D_{F}=\frac{0.00143T^{1.75}}{P{\bar{M}}^{0.5}{\left[{\left(\Sigma_{V}\right)}_{1}^{1/3}+{\left(\Sigma_{V}\right)}_{2}^{1/3}\right]}^{2}}\\ D_{K}=\frac{4}{3}{\left(\frac{8R_{g}T}{\pi M}\right)}^{0.5}\frac{S}{Pe} \end{array}\right.,$$

where *T* denotes absolute temperature, *P* represents ambient pressure, and *Σ_V_* is the diffusion volume of molecules, with values of 18.9 cm^3^/mol for CO, 26.9 cm^3^/mol for CO_2_, and 16.6 cm^3^/mol for O_2_. *M* denotes the molar mass of the gas, *S* represents the cross-sectional area of the crack, *Pe* denotes the perimeter of the crack cross-section, and *R_g_* denotes the gas constant, equal to 8.314 J/(mol·K).

**Figure 9:**
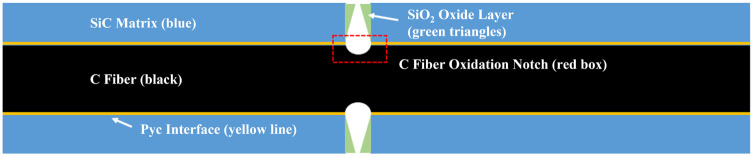
Microstructure of carbon fibre notches.

During the oxidation process, when the mass loss rate of carbon is below 70%, its mass loss exhibits linear growth over time. The rate constant expression for the oxidation behaviour of the carbon phase is defined as [33]:

(3) $$k_{C}=\frac{\Delta m}{m_{0}t},$$

where *Δm* denotes the mass loss of carbon oxidized up to time *t*; *m*_0_ denotes the initial mass. The effect of temperature on the reaction rate can be described by the Arrhenius equation, where the rate constant is expressed as:

(4) $$k_{C}=k_{0}\cdot \exp\left(-E_{r}/R_{g}T\right),$$

where *k*_0_ denotes the pre-exponential factor, and *E_r_* represents the activation energy of the reaction. The activation energy for T700 carbon fibre in an air atmosphere (at temperatures ranging from 900 to 1200 °C) is 12.55 kJ/mol [34]. The fibres within CMCs undergo oxidation primarily at the points of contact with oxygen. Consequently, an oxidation gap forms at the interface with the crack due to this uneven oxidation process, with the resulting morphological changes as shown in Figure 9.

After oxygen diffuses to the fibre surface, it first undergoes oxidation with the interfacial phase. Subsequently, defects propagate both longitudinally and transversely. The reaction rate of the carbon phase can be expressed as [35]:

(5) $$R_{C}=\frac{1}{S_{C}}\frac{n(C)}{dt}=\frac{1}{S_{C}}\frac{S_{C}\rho_{C}dr}{M_{C}dt},$$

where *S_C_* denotes the reaction area, *dr* denotes the reaction length, *ρ_C_* denotes the density of the carbon phase, and *M_C_* denotes the molar mass of carbon. The reaction rate constant for carbon–oxygen reactions is described by the Arrhenius equation in relation to the oxygen concentration at the crack base. The reaction rate of the carbon phase can be expressed as:

(6) $$R_{C}=bk_{C}C_{O_{2}}=bk_{0}\exp\left(-E_{r}/R_{g}T\right)C_{O_{2}},$$

In this equation, *b* denotes the carbon–oxygen reactant consumption ratio, and *C_O_*_2_ represents the oxygen concentration participating in the oxidation reaction. The relationship between the interface thickness and its variation is thus obtained as:

(7) $$r_{I}(t)=2k_{0}^{I}\cdot \exp\left(-E_{r}^{I}/R_{g}T\right)C_{O_{2}}(y,t)\frac{M_{C}}{\rho_{C}}t,$$

where *E_r_^I^* is the reaction activation energy for the interfacial phase (PyC), with a value of 123 kJ/mol [35], while *C_O_*_2_(*y*,*t*) represents the oxygen concentration at the crack tip of the SiC matrix. When the interfacial layer on the fibre surface at the crack site is depleted, oxygen begins to react with the fibre phase through oxidation. Fibre oxidation primarily occurs radially. Assuming the surface reaction rate constant is ten times that at the fibre centre [36], the change in reaction rate can be expressed as:

(8) $$k_{C}^{f}(y)=k_{C}^{f}\left(h_{I}\right)\left[1-\frac{9\left(y-h_{I}\right)}{10R_{f0}}\right],$$

where *R_f_*_0_ denotes the initial fibre radius, while *h_I_* represents the PyC interface thickness, which is taken as 0.5 × 10^−6^ m. Combined with (5), the oxidation rate of the fibrous phase can be expressed as:

(9) $$R_{C}^{f}=2k_{C}^{f}\left(h_{I}\right)\left[1-\frac{9\left(y-h_{I}\right)}{10R_{f0}}\right]C_{O_{2}}(y,t).$$

The consumption of fibres under oxidation is as follows:

(10) $$r_{f}(t)=2k_{C}^{f}\left(h_{I}\right)\left[1-\frac{9\left(y-h_{I}\right)}{10R_{f0}}\right]C_{O_{2}}(y,t)\left(t-t_{f}\right),$$

where *t_f_* denotes the oxidation duration of the interfacial phase. As shown in Figure 9, the fibres at the crack base initiate the reaction first, with their fibre radius expressed as:

(11) $$R_{f}(t)=R_{f0}+h_{I}-r_{f}(t).$$

The strength of fibres diminishes as their diameter decreases due to oxidation, with their load-bearing capacity determined by the remaining effective cross-sectional area. Assuming the fibre material maintains homogeneity during oxidation, the strength of the fibre may be expressed as [35]:

(12) $$\sigma_{f}(t)={\bar{\sigma }}_{f0}\cdot {\left(\frac{R_{f}(t)}{R_{f0}}\right)}^{2},$$

where $\bar{\sigma }$*_f_*_0_ denotes initial fibre strength. Oxygen diffuses through matrix cracks into the composite material, undergoing oxidation reactions with interfaces and fibres at temperatures between 700 and 900 °C. When the temperature exceeds 900 °C, the SiC matrix undergoes predominantly passive oxidation in air, leading to the formation of a protective silica scale. In this work, the SiC oxidation product is treated as SiO_2_, and the silica-scale thickness *δ* is assumed to follow a parabolic growth law [4]:

(13) $$\delta^{2}=Bt=\frac{2D_{SiO_{2}}(T)C_{O_{2}}}{N_{1}}t,$$

(14) $$D_{SiO_{2}}(T)=D_{0}\exp\left(-\frac{Q}{RT}\right),$$

where *B* is the parabolic rate constant, *D_SiO_*_2_(*T*) is the oxygen diffusion coefficient in SiO_2_, *N*_1_ denotes the molar concentration of the oxide (i.e., the number of moles of SiO_2_ per unit volume of the silica scale), which is evaluated as *N*_1_ = *ρ_SiO_*_2_*/M_SiO_*_2_, and *D*_0_ and *Q* are the Arrhenius prefactor and activation energy for molecular oxygen diffusion through the silica scale (SiO_2_), respectively; their values are listed in Table 1. Following the classical diffusion-controlled oxidation assumption, *B* can be expressed in terms of oxygen transport through SiO_2_. When SiC undergoes passive oxidation reactions, the volume of SiO_2_ produced is not equal to the volume of SiC consumed due to the difference in the nature of the constituent substances, but the following proportionality exists:

(15) $$V_{r}=\frac{V_{{\mathrm{SiO}}_{2}}}{V_{\mathrm{SiC}}}=\frac{\frac{n_{{\mathrm{SiO}}_{2}}M_{{\mathrm{SiO}}_{2}}}{\rho_{{\mathrm{SiO}}_{2}}}}{\frac{n_{\mathrm{SiC}}M_{\mathrm{SiC}}}{\rho_{\mathrm{SiC}}}}=\frac{M_{{\mathrm{SiO}}_{2}}\rho_{\mathrm{SiC}}}{M_{\mathrm{SiC}}\rho_{{\mathrm{SiO}}_{2}}},$$

where *V_r_* denotes the ratio of oxidation product to reactant volume, *V_SiO_*_2_, *V_SiC_* denote the volume of each of the two materials, *M_SiO_*_2_, *M_SiC_* denote the molar mass of each of the materials, and *ρ_SiO_*_2_, *ρ_SiC_* denote the density of each of the two materials. The developing SiO_2_ scale further affects oxygen ingress by progressively narrowing the crack transport channel (silica filling and crack-wall thickening). To account for this coupling, the effective crack opening *e*(*t*) is updated by considering thermal mismatch, applied stress, and oxide growth [37]:

(16) $$e(t)=\frac{e_{0}}{T_{0}}\left[\Delta T+\frac{1}{E_{f}V_{m}\left(\alpha_{m}-\alpha_{f}\right)}\sigma \right]-2\Delta \delta ,$$

(17) $$\Delta \delta =\delta \frac{V_{r}-1}{V_{r}},$$

where *e*_0_ is the crack width of the matrix at room temperature, *T*_0_ is the material preparation temperature, *σ* is the external load, *α_m_* is the thermal expansion coefficient of the matrix, *α_f_* is the thermal expansion coefficient of the fibres, *E_f_* is the elastic modulus of the fibres, *V_m_* is the volume content of the matrix, and Δδ is the change in thickness due to oxidation of the matrix. In this work, it is assumed that the actual area of the SiC reaction is equal to the area of the SiO_2_ produced, and the thickness ratio of the two is expressed using their volume ratio (Equation (17)). The time-dependent crack opening *e*(*t*) is used to update the crack geometric terms *S* and *Pe* in Equation (2), thereby updating *D_K_* and the equivalent diffusion coefficient *D* throughout oxidation exposure.

Based on the molar flux continuity equation, the molar flux expression within the material is derived as [4]:

(18) $$\nabla N_{O_{2}}+\frac{\partial C_{O_{2}}(y,t)}{\partial t}+R_{O_{2}}=0,$$

where *N_O_*_2_ represents the gradient of the oxygen molar flux, *C_O_*_2_(*y*,*t*) denotes the oxygen concentration at time *t* and crack depth *y*, and *R_O_*_2_ is the chemical reaction consumption rate of oxygen. In the present 1D crack-depth model, $\nabla N_{O2}$ reduces to $\partial N_{O2}/\partial y$. Under isothermal and isobaric conditions, the oxygen diffusion flux can be expressed as:

(19) $$N_{O_{2}}(y,t)=\frac{-D_{O_{2}}(y,t)}{1+\chi_{O_{2}}(y,t)}\frac{\partial C_{O_{2}}(y,t)}{\partial y},$$

where *D_O_*_2_ represents the diffusion coefficient of oxygen and *χ_O_*_2_(*y*,*t*) denotes the molar fraction of oxygen, and can also be expressed as:

(20) $$\chi_{O_{2}}(y,t)=\frac{C_{O_{2}}(y,t)}{C_{C}},$$

where *Cc* represents the initial oxygen concentration, calculated using the ideal gas equation. The SiO_2_ formed by oxidation of the matrix can be expressed as:

(21) $$\frac{\partial n_{{\mathrm{SiO}}_{2}}}{\partial t}=\frac{\rho_{{\mathrm{SiO}}_{2}}S_{{\mathrm{SiO}}_{2}}}{M_{{\mathrm{SiO}}_{2}}}\frac{\partial \delta (y,t)}{\partial t}=\frac{\rho_{{\mathrm{SiO}}_{2}}S_{{\mathrm{SiO}}_{2}}B(y,t)}{2M_{{\mathrm{SiO}}_{2}}\delta (y,t)},$$

In this equation, *S_SiO_*_2_ represents the surface area of the SiO_2_ generated by oxidation. According to the passive oxidation equation, the molar ratio of reactants to oxidation products in the oxidation reaction satisfies a 3:2 relationship. Therefore, along the crack depth y, the amount of O_2_ consumed per unit time t on the inner wall of the crack per unit length is as follows:

(22) $$\frac{\partial n_{O_{2}}(y,t)}{\partial t}=\frac{3}{2}\frac{\partial n_{{\mathrm{SiO}}_{2}}(y,t)}{\partial t}=\frac{3\rho_{{\mathrm{SiO}}_{2}}S_{{\mathrm{SiO}}_{2}}B(y,t)}{4M_{{\mathrm{SiO}}_{2}}\delta (y,t)}.$$

At time *t* and location *y* in the CMCs, the differential equation governing the oxidation kinetics is as follows [36]:

(23) $$R_{O_{2}}(y,t)=2\frac{\partial n_{O_{2}}(y,t)}{\partial y\cdot \partial t}=\frac{3\rho_{{\mathrm{SiO}}_{2}}lB(y,t)}{2M_{{\mathrm{SiO}}_{2}}\delta (y,t)},$$

Ignoring the effects of oxygen flow velocity and the boundary layer on the material surface, the oxygen concentration at the crack entrance is considered equivalent to the ambient value. At the crack base, diffusion is limited by the consumption rate due to the chemical reaction between oxygen and carbon, thereby establishing the boundary conditions for the differential equation governing the oxidation process.

(24) $$\left\{\begin{array}{c} y=0,C_{O_{2}}=C_{C}\\ y=L,\frac{-D_{O_{2}}(L,t)S(L,t)}{1+\chi_{O_{2}}(L,t)}\frac{\partial C_{O_{2}}(L,t)}{\partial y}=\frac{k_{0}\rho_{C}V_{f}V_{C}}{2M_{C}}{\left(\frac{C_{O_{2}}(L,t)}{C_{0}}\right)}^{P_{C}} \end{array}\right.,$$

In this equation, *V_f_* represents the fibre volume fraction, and *V_c_* is the volume of the composite test section. *V_c_ = L*_0_*W*_0_*H*_0_, where *L*_0_ is taken as the effective exposed gauge length of the specimen, *W*_0_ and *H*_0_ denote the characteristic dimensions of a representative oxygen-transport microcrack opening (i.e., the microcrack aperture) used to define the oxidation-kinetics control volume. At any given time *t*, the oxidation kinetics satisfy the aforementioned equations. By transforming the boundary value problem into an initial value problem, a numerical solution is obtained. Through iterative computation, the concentration distribution as a function of time is ultimately derived.

**Figure 10:**
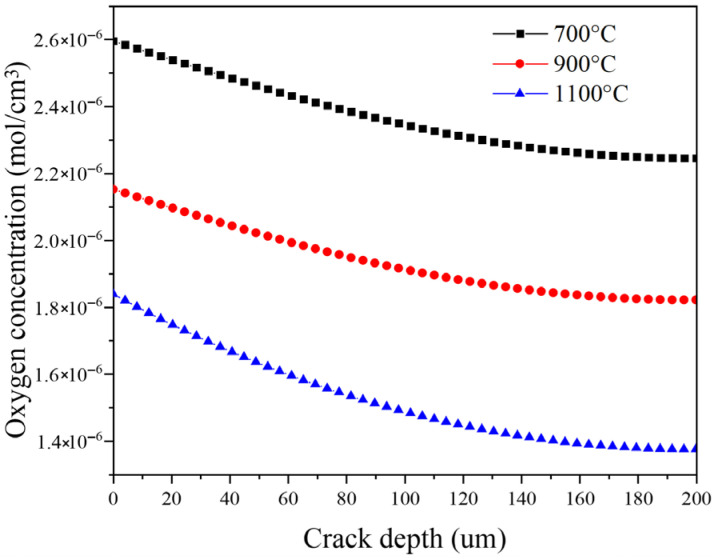
Oxygen concentration profiles along the crack depth at different temperatures, obtained by numerically solving Equations (18)–(20), (23) and (24).

The oxygen concentration distribution along the crack depth exhibits a monotonic decline after 1000 s of oxidation exposure, as illustrated in Figure 10. The profiles in Figure 10 were obtained by numerically solving the 1D diffusion–reaction problem along the crack depth, where *C_O_*_2_(*y*,*t*) is computed from the molar-flux continuity equation (Equation (18)) together with the diffusion-flux relations (Equations (19) and (20)); the chemical consumption is introduced through the reaction term (Equation (23)) and the boundary conditions at the crack mouth and crack base are prescribed in Equation (24). This gradient results from the competing effects of oxygen diffusion and its consumption through the formation of SiO_2_. Near the crack mouth, the concentration remains close to the external oxygen level, indicating unobstructed diffusion. However, with increasing depth, the diffusional resistance imposed by the narrow crack geometry becomes pronounced, resulting in a progressively steeper concentration decay. The higher temperatures (900 °C and 1100 °C) further intensify this gradient. This trend reflects the accelerated oxidation kinetics at elevated temperatures: the rapid formation of silica scale causes local crack-wall thickening, effectively reducing the crack aperture and hindering further oxygen ingress. Consequently, oxygen availability at the crack tip becomes more limited at higher temperatures, indicating a progressive shift from diffusion-controlled to reaction-limited oxidation as crack closure develops.

**Figure 11:**
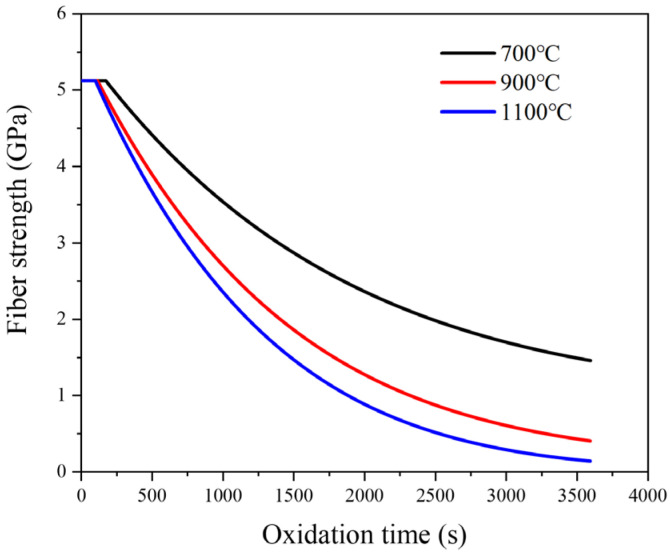
Evolution of fibre strength with oxidation time at 700, 900 and 1100 °C (air).

As shown in Figure 11, the predicted evolution of fibre strength versus oxidation time (*t*, in seconds) at 700, 900, and 1100 °C is depicted. The strength-loss rate increases from 700 °C to 900 °C due to the thermally activated carbon-oxidation kinetics. When the temperature exceeds 900 °C, concomitant passive oxidation of the SiC matrix leads to SiO_2_ scale formation, which progressively narrows the crack transport channel and reduces the effective oxygen ingress. This diffusion-limiting effect (implemented through the SiO_2_ growth and the updated oxygen transport terms in Section 4.1) mitigates the apparent acceleration from 900 °C to 1100 °C within the studied range. The present formulation is applied only to the experimental temperatures (700–1100 °C) and is not intended for extrapolation beyond this range.

### 4.2. Damage to Material Constituents Caused by Oxidation

During the manufacturing process of carbon fibre, numerous randomly distributed defects inevitably arise. The actual strength of the fibre is determined by these randomly distributed defects, and its ultimate strength is dictated by the distribution of its weakest defects. To describe the statistical strength of carbon fibres, this work adopts a discrete empirical distribution (histogram-based empirical distribution) following the approach in Ref. [39]. From Ref. [40], a dataset of 250 single-filament tensile strengths measured at room temperature with a gauge length of 25 mm was collected, and the strengths were grouped into 40 discrete levels *σ_i_* (1 ≤ *i* ≤ 40). Each fibre is discretized into *N* uniformly spaced microelements, and defects on different microelements are assumed to be mutually independent.

Using event *A_i_* to denote the presence of a defect of level *i* on the fibre and event $A_{i}^{j}$ to denote the presence of a defect of level *i* on the *j-th* infinitesimal segment of the fibre, it is evident that events *A_i_* and $A_{i}^{j}$ are related as follows:

(25) $$A_{i}=\underset{j}{\cup }A_{i}^{j},P(A_{i})=P\left(\underset{j}{\cup }A_{i}^{j}\right)=l-P\left(\underset{j}{\cap }\bar{A_{i}^{j}}\right)=1-\prod_{j}\left[1-P(A_{i}^{j})\right].$$

using *B_i_* to denote the strength of the fibre as *σi*. When event *B_i_* occurs, there exists no microelement on the fibre with a defect grade lower than the *i-th* grade. Therefore, events *A_i_* and *B_i_* are related as follows:

(26) $$P(B_{i})=P(A_{i})\cdot \prod_{k=1}^{i-1}\left[1-P(A_{k})\right].$$

**Figure 12:**
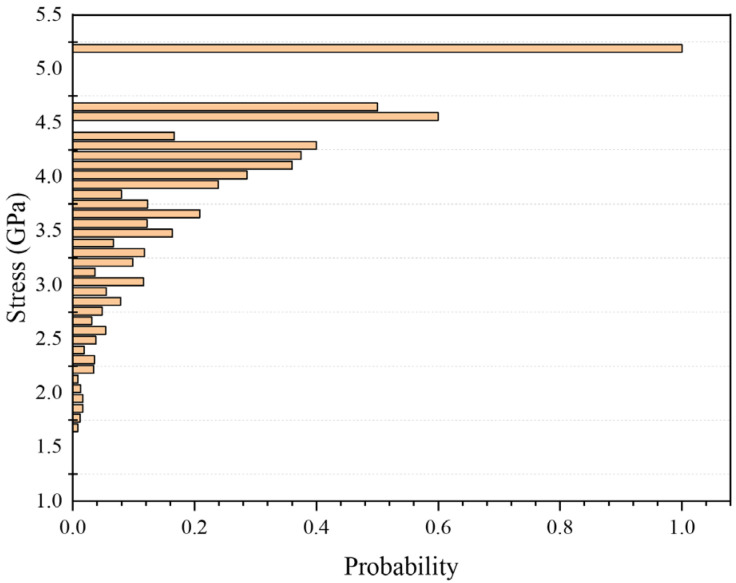
Probability distribution of defects in carbon fibre grades.

The probability of event *B_i_* occurring is denoted as *P*(*B_i_*), representing the ratio of the number of fibres with strength *σi* to the total number of fibres in the sample. By obtaining the strength and quantity corresponding to each defect grade and calculating *P*(*B_i_*), the statistical results are shown in Appendix A, Table A1. This treatment is consistent with the weakest-link concept widely used for brittle fibres, where the filament strength is governed by the most critical defect along its length [41]. The probability *P*(*A_i_*) of the *i-th* defect grade being present on a fibre can be determined using Formula (27). The results are illustrated in Figure 12.

(27) $$P(A_{i})=\frac{P(B_{i})}{\prod_{k=1}^{i-1}\left[1-P(A_{k})\right]}.$$

Since each infinitesimal element possesses identical and uniform lengths, the probability of a defect of the *i-th* order occurring on any infinitesimal element is equal and can be expressed as:

(28) $$P(A_{i}^{1})=P(A_{i}^{j})=P(A_{i}^{N})=P_{i}.$$

Substituting Equation (28) into Equation (25) yields the probability *P_i_* that a defect of the *i-th* order exists on the infinitesimal element:

(29) $$P(A_{i})=1-{\left(1-P_{i}\right)}^{N}\Rightarrow P_{i}=1-{\left[1-P)A_{i})\right]}^{1/N}.$$

Upon obtaining *P_i_*, the strength of each microelement may be allocated using a random number method. Generate a random number *n_s_* (0 < *n_s_* < 1). If *n_s_* < *P_i_*, assign the corresponding defect to this microelement. The strength *σ_∆__l_* of this microelement shall be the strength *σi* of the corresponding defect. Defect propagation during oxidation reduces the filament strength; therefore, the initial grade *i_in_* shifts toward a lower-strength grade *i_oxi_* after oxidative damage. The grade shift is quantified by the reduction in maximum filament strength:

(30) $$\Delta \sigma ={\bar{\sigma }}_{f0}-\sigma_{oxi}.$$

where $\bar{\sigma }$*_f_*_0_ is the initial maximum strength of the fibre (this value is 5.19 GPa from Appendix A, Table A1) and *σ_oxi_* is the initial maximum strength of the fibre after oxidation. From the calculations in Section 4.1, the expression for the update of the defect grade of each microelement is Equation (32) and the expression for the update rule of the defect distribution is Equation (33). This means that microelements that are initially at rank *i_in_* will migrate to rank *i_oxi_* with probability *P’*(*A_i_*) after oxidation.

(31) $$\Delta i=\mathrm{round}\left(\frac{\Delta \sigma }{\Delta \sigma_{\mathrm{grade}\;}}\right),\;\Delta \sigma_{\mathrm{grade}\;}=\frac{\sigma_{40}-\sigma_{1}}{39}.$$

(32) $$i_{oxi}=i_{in}-\Delta i.$$

(33) $$P^{\prime }(A_{i})=P(A_{i}).$$

**Figure 13:**
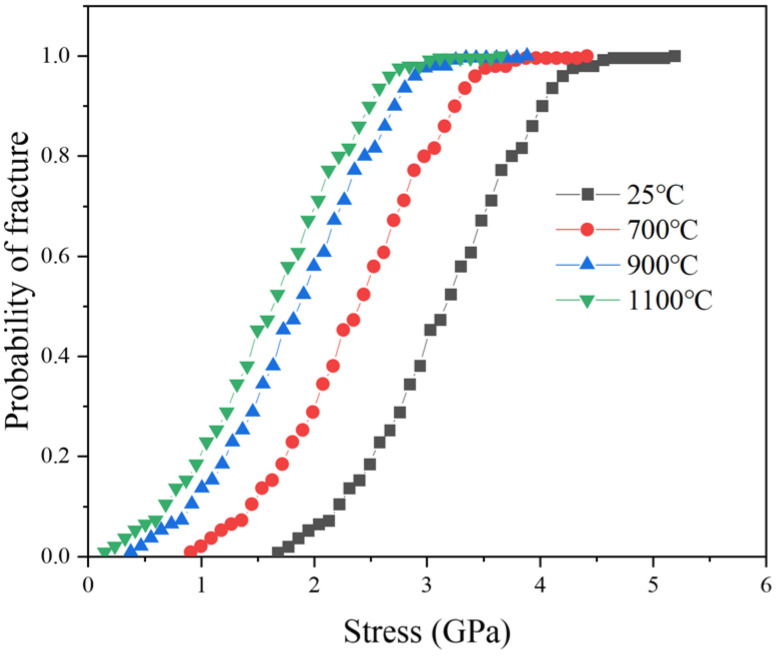
Distribution of carbon fibre strength under different oxidation temperature conditions.

Here, *i_oxi_* is truncated to the interval [1, 40] to remain within the defined strength grades; the minus sign reflects the strength degradation (i.e., oxidation drives the distribution toward lower-strength grades. Based on the calculated oxidation-degraded fibre strength, the fracture force of the carbon-fibre bundle was evaluated. Using the bundle fracture criterion, the fibre strength distribution was then equivalently shifted for each oxidation temperature, yielding the temperature-dependent distributions shown in Figure 13.

When CMC materials are subjected to tensile loading, cracks within the matrix commence propagation. The interface causes deflection of the crack path, thereby transferring the load borne by the fibres through the interface zone to the matrix. This achieves stress redistribution, preventing premature fibre failure and enhancing toughness. During this process, debonding and slip phenomena occur at the fibre/matrix interface. The maximum shear stress criterion is employed as the debonding criterion for the interface, expressed as follows [42]:

(34) $$\tau_{i}\geq \tau_{\mathrm{ult}},$$

where *τ_i_* represents the shear stress of the interfacial phase and *τ_ult_* is the critical shear stress of the interfacial phase. According to the shear lag model, the stress distribution on the fibre is as follows:

(35) $$\sigma_{f}(x)=\left\{\begin{array}{c} \sigma_{f0},0\leq x\leq \frac{L}{2}-d_{f}\\ \frac{2\tau_{i}}{R_{f}}\left(x-\frac{L}{2}\right)+\frac{\sigma }{V_{f}},\frac{L}{2}-d_{f}\leq x\leq \frac{L}{2} \end{array}\right.,$$

where *σ* represents the external load acting on both ends of the fibre bundle composite material. *d_f_* denotes the slip distance. *σ_f_*_0_ is the normal stress in the bonded zone, numerically equivalent to the fibre stress at matrix fracture when no interfacial debonding has occurred.

(36) $$\left\{\begin{array}{c} \sigma_{f0}=\frac{E_{f}}{E_{C}}\sigma +E_{f}\left(\alpha_{C}-\alpha_{f}\right)\Delta T\\ E_{C}=V_{f}E_{f}+V_{m}E_{m}\\ \alpha_{C}=\frac{\alpha_{f}V_{f}E_{f}+\alpha_{m}V_{m}E_{m}}{E_{C}} \end{array}\right.,$$

where *α_c_* denotes the coefficient of thermal expansion of the composite material, *E_c_* represents the elastic modulus of the composite material, *E_m_* is the elastic modulus of the matrix, and *∆T* is the difference between the preparation temperature of the composite material and the ambient temperature.

The application of additional stress not only affects the width of the initial matrix crack but also promotes the formation of entirely new cracks within the matrix. This article employs the Critical Matrix Strain Energy (CMSE) criterion, whose mathematical expression is [43]:

(37) $$U_{m}=U_{cr},$$

In this equation, *U_m_* is the matrix strain energy, which can be calculated using the following formula:

(38) $$U_{m}=\int_{V}\int_{S}\sigma_{m}(x)d\varepsilon dV.$$

When subjected to loading, the average stress across different cross-sections of CMCs remains constant. By combining the fibre stress distribution within the slip zone and the bond zone [44], the stress distribution within the matrix at different cross-sections can be calculated:

(39) $$\sigma (x)=\left\{\begin{array}{c} \frac{-2V_{f}\tau_{i}}{V_{m}R_{f}}\left(x-\frac{L}{2}\right),\frac{L}{2}-d_{f}\leq x\leq \frac{L}{2}\\ \sigma_{m0} \end{array}\right.,$$

In this equation, *σ_m_*_0_ represents the normal stress of the matrix in the bonded zone, which can similarly be calculated using the mixing ratio formula:

(40) $$\sigma_{m0}=\frac{E_{m}}{E_{C}}\sigma +E_{m}(\alpha_{C}-\alpha_{m})\Delta T.$$

Substituting Equations (39) and (40) into Equation (38) yields:

(41) $$U_{m}=\frac{A_{m}}{V_{m}^{2}R_{f}^{2}E_{m}}[\frac{1}{2}{\left(V_{m}R_{f}\sigma_{m0}\right)}^{2}(L-2d_{f})+\frac{4}{3}{\left(\tau_{i}V_{f}d\right)}^{2}d_{f}].$$

The crack spacing L in the matrix may be calculated using the CMSE guideline via Equation (41).

### 4.3. Stress–Strain Calculation

During loading, fibres fracture sequentially. At the crack plane within the matrix, interfacial shear stress forms an “influence zone’’. Fibre fractures within this zone redistribute stress at the matrix crack without affecting fibres outside the zone. The length of the influence zone can be expressed as [45]:

(42) $$L_{ef}=\frac{\sigma R_{f}}{V_{f}\tau_{i}}.$$

On the basis of the stress at the fibre in the matrix crack, the average stress of the material can be expressed as:

(43) $${\bar{\sigma }}_{C}=\left(\frac{1}{N}\sum_{i=1}^{N_{f}}\frac{2\tau_{i}}{R_{f}}L_{b_{i}}+\frac{N-N_{\Delta l}}{N}\frac{\sigma }{V_{f}}\right)V_{f},$$

In this equation, *N* denotes the total number of fibre monofilaments, *N_Δ__l_* denotes the number of fibre monofilaments within the “affected zone’’, and *L_bi_* denotes the distance from the internal fibre within the matrix where fracture occurs to the matrix crack. The average strain of a single intact, unbroken fibre within the material constitutes the material’s average strain, as follows:

(44) $${\bar{\varepsilon }}_{C}={\bar{\varepsilon }}_{f}=\frac{\Sigma \Delta L}{\Sigma L},$$

In this equation, *L* denotes the inter-crack spacing within the matrix, which may be calculated using the CMSE criterion from Equation (41). *ΔL* represents the deformation of unfractured fibres within adjacent matrix cracks, which may be determined by calculating the fibre stress distribution as follows:

(45) $$\Delta L=\frac{2}{E_{f}}\int_{0}^{\frac{L}{2}}\sigma_{f}(x)dx+\left(\alpha_{f}-\alpha_{C}\right)\Delta TL.$$

**Figure 14:**
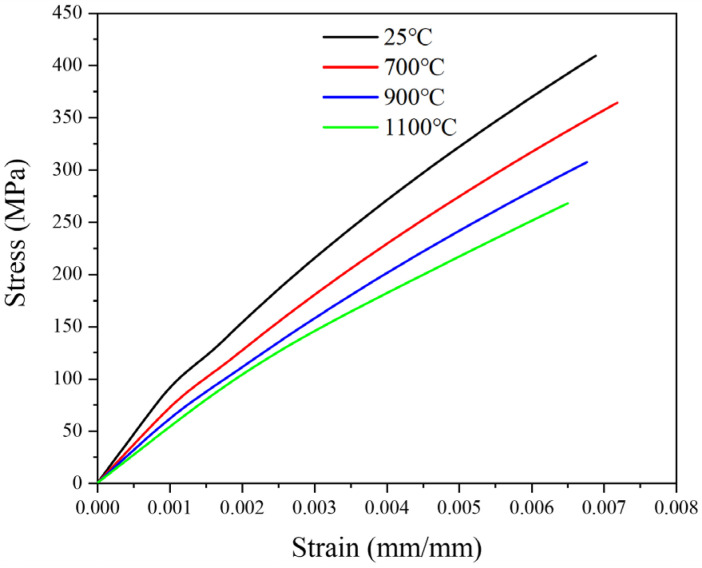
Predicted stress–strain response of yarn.

A detailed examination of the constitutive model material parameters is presented in Table 2. According to the aforementioned oxidation constitutive response calculation method, tensile stress–strain curves for yarns at different oxidation temperatures can be obtained, as shown in Figure 14.

## 5. Model Parameters and Calculation Results

**Figure 15:**
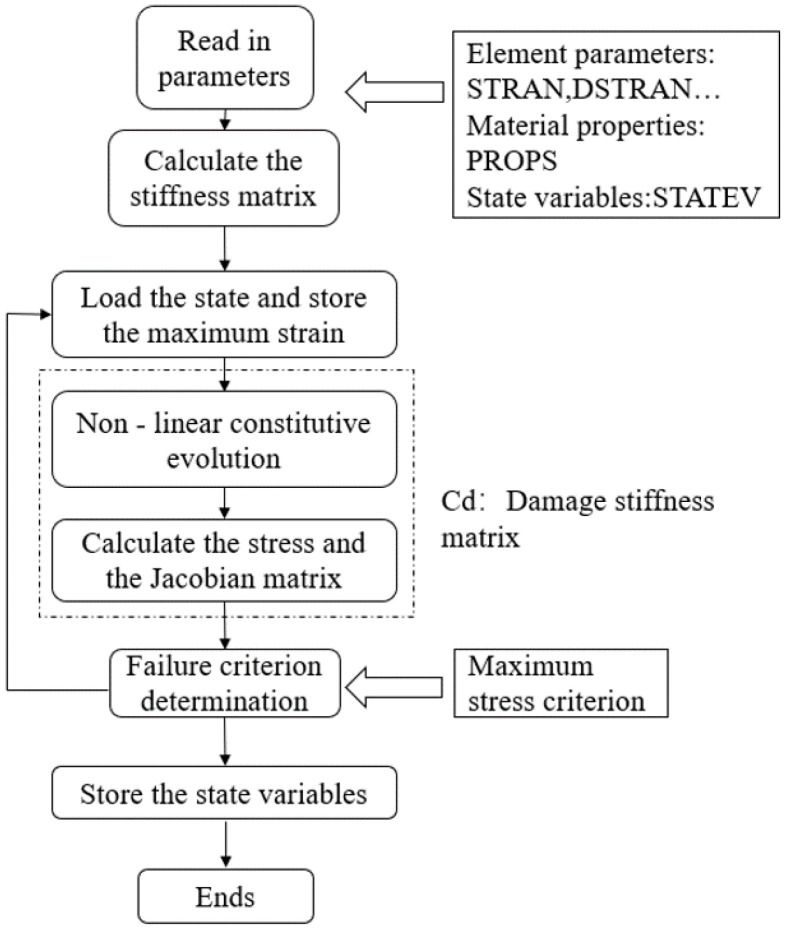
UMAT computation process.

Using a yarn oxidation constitutive model, the nonlinear oxidation constitutive behaviour of woven C/SiC composites is described via the ABAQUS-USERMAT functionality. The computational flowchart for the USERMAT subroutine is presented in Figure 15. When considering the effects of oxidative damage, the modulus of CMCs during the reloading phase of the loading–unloading process can be expressed as [36]:

(46) $$E_{C}^{\prime }=E_{C}\cdot (1-D_{ox}),$$

In this equation, *E′_C_* denotes the modulus of the reloading segment, and *D_ox_* is the CMC oxidation damage coefficient. *D_ox_* is evaluated as:

(47) $$D_{ox}(T)=m_{1}{\left(1-\exp{\left(-\frac{T}{T_{0}}\right)}^{m_{2}}\right)}^{m_{3}}+m_{4},$$

where *m*_1_–*m*_4_ are the fitting parameters; *T*_0_ is a reference temperature (K) used for normalization (*T*_0_ = 1473 K). Here, *D_ox_* is used as an effective reloading-modulus reduction coefficient to account for the temperature-dependent degradation observed during cyclic loading–unloading. At room temperature, where oxidation is negligible, *D_ox_* mainly reflects the baseline cyclic damage (e.g., matrix microcracking and interfacial sliding). The additional reduction observed at elevated temperatures is attributed to oxidation-assisted degradation mainly in the matrix and fibre/matrix interface. Equation (47) is introduced as a phenomenological closure for this temperature dependence; it is applied only to update the reloading modulus in Equation (46) and does not alter the physics-based fibre oxidation kinetics or the failure criterion. The parameters *m*_1_–*m*_4_ were identified by least-squares fitting to the discrete *D_ox_* values extracted from the experimental cyclic curves (Table 3), with the bounds 0 ≤ *D_ox_* ≤ 1.

**Figure 16:**
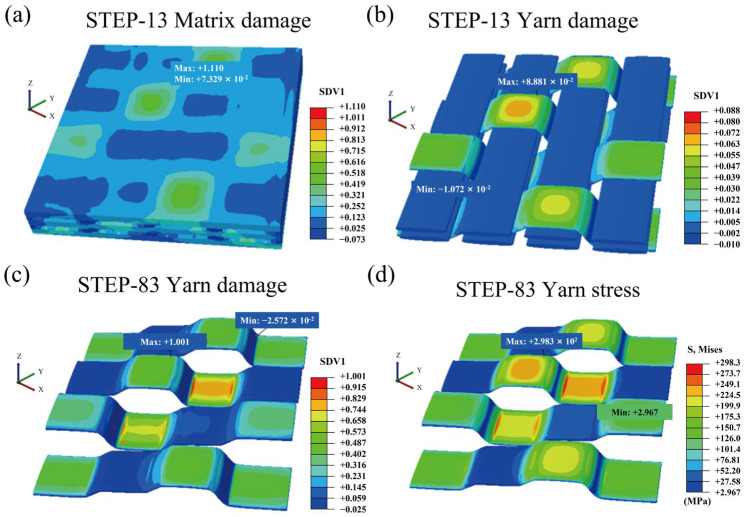
Damage development and stress contour diagram. (**a**) STEP-13 matrix damage; (**b**) STEP-13 yarn damage; (**c**) STEP-83 yarn damage; (**d**) STEP-83 yarn stress.

Based on the mechanical properties of yarns under different oxidation temperatures, a progressive damage approach was employed to conduct tensile calculations for 2.5D woven C/SiC composites. Typical damage evolution and stress contour plots are illustrated in Figure 16. In this work, damage in the material direction *1* is defined by the state variable STATEV(1), effectively characterizing the damage evolution of each component during progressive loading. Results indicate that during the initial loading stage (STEP-13), matrix damage rapidly develops and reaches a maximum value of 1, signifying complete failure. At this point, the maximum fibre damage value is merely 0.08, remaining within the elastic range without significant damage occurrence. This demonstrates that the fibres, acting as the primary load-bearing units, effectively delayed the overall failure of the material. Considering that the warp fibres bear the main load in the 2.5D woven C/SiC composites, their damage and stress contour plots were further extracted for analysis. Results indicate that during the late loading stage (STEP-83), fibre damage gradually accumulated and ultimately reached 1, causing the entire material to enter a failure state, thereby terminating the calculation.

**Figure 17:**
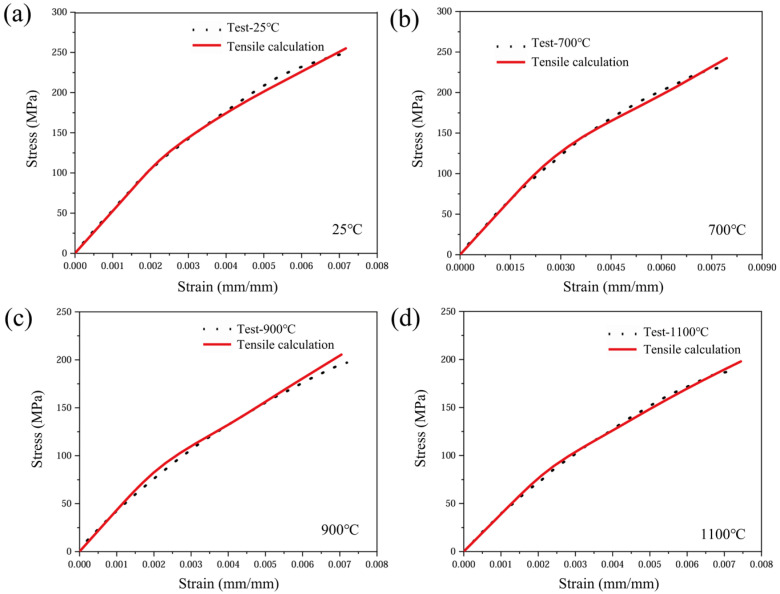
Predicted oxidation constitutive behaviour of 2.5D woven C/SiC composites under varying temperature conditions. (**a**) 25 °C; (**b**) 700 °C; (**c**) 900 °C; (**d**) 1100 °C.

The test results are shown in Figure 17. To validate the model’s validity and accuracy, the figure also presents the predicted strain response curve for the axial tensile model. The 2.5D woven C/SiC composites exhibit pronounced temperature dependence across three distinct stages during tensile testing. As the temperature rises to 700 °C and above, oxidation causes a decrease in the elastic modulus of the material components, resulting in a reduced slope for the first linear segment. The second nonlinear segment exhibits an earlier onset with increasing temperature, accompanied by more pronounced nonlinear behaviour. This phenomenon is particularly evident at 900 °C and 1100 °C. The second linear segment is significantly influenced by the reduced elastic modulus and strength of oxidized fibres. At 25 °C and 700 °C, the fibres remain capable of effectively bearing loads, exhibiting a relatively stable stress–strain relationship. But at 900 °C and 1100 °C, oxidative damage leads to fibre fracture and interfacial delamination, markedly diminishing the material’s load-bearing capacity. Consequently, the linear segment of the curve shortens and strength diminishes.

**Figure 18:**
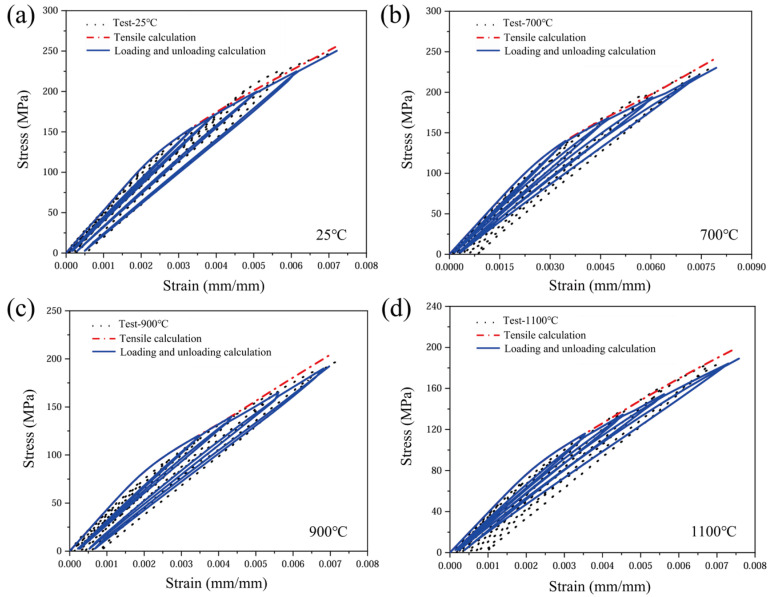
Predicted results of tensile stress–strain response for 2.5D woven C/SiC composites under different oxidation temperatures. (**a**) 25 °C; (**b**) 700 °C; (**c**) 900 °C; (**d**) 1100 °C.

Considering the effects of oxidative damage, the material modulus during the counterweight loading phase of the CMCs was calculated using a reduction factor during the loading and unloading process, resulting in the tensile loading and unloading curve shown in Figure 18. The loading–unloading stress–strain curves of 2.5D woven C/SiC composites are similarly influenced by the internal yarn mechanical response within the material. The stress–strain curves of 2.5D woven C/SiC composites reveal pronounced hysteresis loops during loading and unloading phases, alongside modulus reduction during reloading. This behaviour is corroborated by the computational results of the stress–strain curves. However, due to material dispersion, the impact of the test environment on high-temperature strain measurement, and the complex interlayer connection structure of woven CMCs, the calculated hysteresis loop exhibits relatively significant errors.

## 6. Conclusions

To elucidate the oxidation-driven degradation mechanisms of 2.5D woven C/SiC composites and to support reliable stiffness evaluation and structural design, this study developed an oxidation-coupled constitutive framework grounded in gas-diffusion behaviour and component-level oxidation kinetics.

The main findings can be summarized as follows:

1. The evolution of fibre strength under oxidation was quantified by integrating intrinsic defect statistics with temperature-dependent oxidation kinetics. A shifted defect-distribution model enabled the estimation of fibre fracture probability across temperatures, while the coupling of fibre/matrix interfacial slip behaviour with a matrix energy-release criterion allowed the tensile response of yarns to be captured with improved physical fidelity. This provides a mechanistic description of how oxidation progressively impairs load transfer at the microscale.

2. RVE models reconstructed from X-CT scans successfully transferred the yarn-scale constitutive behaviour into a mesostructural representation of the woven architecture. By embedding oxidation-induced stiffness loss into the finite-element asymptotic damage formulation, the tensile failure process of the composite at elevated temperatures was reproduced with good agreement with experimental observations. The modelling strategy accurately captured both the degradation of the elastic modulus and the nonlinear unloading–reloading characteristics arising from oxidation-induced microstructural evolution.

Overall, the proposed oxidation-coupled constitutive framework establishes a mechanistic link between fibre oxidation, yarn load transfer, and composite-scale stiffness degradation. It provides a practical basis for evaluating the residual mechanical performance of C/SiC composites in oxidizing environments, and can in principle be extended to other oxidation durations and to cyclic loading by driving the oxidation-transport module with the desired exposure time and the constitutive update with a prescribed stress history (with an additional fatigue-damage law introduced if long-life prediction is required), which will be addressed in future work.

## Figures and Tables

**Table 1:** Oxidation kinetics parameters of C/SiC composites.

Parameter	Symbol	Value	Unit	Source
Representative exposure length along oxygen transport direction (crack depth)	*L*_0_	0.15	m	Specimen gauge (this work)
Characteristic microcrack width (transport channel)	*W*_0_	1.02 × 10^−6^	m	Estimated from micrographs (this work)
Characteristic microcrack height (transport channel)	*H*_0_	3.3 × 10^−6^	m	Estimated from micrographs (this work)
Intact fibre radius	*R_f_*_0_	2.94 × 10^−6^	m	Estimated from micrographs (this work)
PyC interphase thickness	*h_I_*	0.5 × 10^−6^	m	Process specification (this work)
O_2_ diffusivity in SiO_2_ (Arrhenius prefactor)	*D*_0_	2.0 × 10^−13^	m^2^ s^−1^	Literature (citation: Ref. [38])
O_2_ diffusivity in SiO_2_ (activation energy)	*Q*	121.3	kJ mol^−1^	Literature (citation: Ref. [38])
Molar concentration of the oxide	*N*_1_	3.66 × 10^4^	mol m^−3^	Computed from *ρ_SiO_*_2_, *M_SiO_*_2_
Molar mass of SiO_2_	*M_SiO_*_2_	60.084	g mol^−1^	Standard chemical constant (CRC/NIST)
Molar mass of SiC	*M_SiC_*	40.096	g mol^−1^	Standard chemical constant (CRC/NIST)
Density of SiO_2_	*ρ_SiO_*_2_	2.20	g cm^−3^	Technical datasheet (Nikon NIFS)
Density of SiC	*ρ_SiC_*	3.21	g cm^−3^	Manufacturer datasheet (supplier-provided)

**Table 2:** Microscopic parameters of yarn.

Parameters	Values
*E_f_*	406 GPa
*E_m_*	230 GPa
*L*	0.0041 m
*α* *_f_*	−0.0156 × 10^−6^
*α* *_m_*	2.2 × 10^−6^
*T*	1200 °C

**Table 3:** Identification of *D_ox_* from cyclic loading–unloading tests and fitted parameters in Equation (47).

Category	Temperature/Parameter	*E_c_* from Upper Envelope (GPa)	*E_c_′* from Reloading (GPa)	*D_ox_*
Extracted from tests	25 °C	111.58	78.76	0.294
	700 °C	99.64	63.40	0.364
	900 °C	84.65	53.01	0.374
	1100 °C	41.28	25.52	0.382
Fitted parameters in Equation (47)	*m*_1_	0.405	-	-
	*m*_2_	1.771	-	-
	*m*_3_	0.112	-	-
	*m*_4_	0.000	-	-

Note: *T* and *T*_0_ are in Kelvin in Equation (47).

## Data Availability

The original contributions presented in this study are included in the article. Further inquiries can be directed to the corresponding authors.

## Abbreviations

The following abbreviations are used in this manuscript:

CMCs	Ceramic Matrix Composites
PIP	Polymer Impregnation and Pyrolysis
CVI/CVD	Chemical Vapour Infiltration/Deposition
SEM	Scanning Electron Microscope
PyC	Pyrolytic Carbon
CMSE	Critical Matrix Strain Energy
RVE	Representative Volume Element
UMAT	User Material

## Appendix A

**Table A1:** Strength grading of defects in carbon-fibre monofilaments.

Level	*σ_i_* (GPa)	Quantity (Count)	*P*(*B_i_*)	Level	*σ_i_* (GPa)	Quantity (Count)	*P*(*B_i_*)
1	1.68	2	0.008	21	3.48	16	0.064
2	1.77	3	0.012	22	3.57	10	0.040
3	1.86	4	0.016	23	3.66	15	0.060
4	1.95	4	0.016	24	3.75	7	0.028
5	2.04	3	0.012	25	3.84	4	0.016
6	2.13	2	0.008	26	3.80	11	0.044
7	2.22	8	0.020	27	4.02	10	0.040
8	2.31	8	0.022	28	4.11	9	0.035
9	2.40	4	0.016	29	4.20	6	0.024
10	2.49	8	0.032	30	4.29	4	0.016
11	2.58	11	0.044	31	4.38	1	0.004
12	2.67	6	0.024	32	4.47	0	0.000
13	2.76	9	0.036	33	4.56	3	0.012
14	2.85	14	0.056	34	4.55	1	0.004
15	2.94	9	0.036	35	4.74	0	0.000
16	3.03	18	0.072	36	4.83	0	0.000
17	3.12	5	0.020	37	4.82	0	0.000
18	3.21	13	0.052	38	5.01	0	0.000
19	3.30	14	0.056	39	5.10	0	0.000
20	3.39	7	0.028	40	5.19	1	0.004

**Table A2:** Nomenclature.

Symbol	Physical Quantity	Symbol	Physical Quantity
*D_F_*	Fick diffusion coefficient	*T*	Absolute temperature
*P*	Ambient pressure	*Σ* *_V_*	Molecular diffusion volume
*M*	Molar mass	*D_k_*	Knudsen diffusion coefficient
*S*	Crack surface area	*Pe*	Perimeter of crack cross-section
*R_g_*	Gas constant	*D_eff_*	Equivalent diffusion coefficient
*k_C_*	Carbon-phase oxidation rate	*k*_0_	Pre-exponential factor
*E_r_*	Reaction activation energy	*E_r_^f^*	Activation energy for carbon fibre reaction
*R_C_*	Carbon-phase reaction constant	*ρ_C_*	Density of carbon phase
*b*	Carbon reaction stoichiometric constant	*C_O_*_2_	Oxygen concentration
*t_f_*	Oxidation duration at fibre/matrix interface	*R_f_*_0_	Initial radius of fibre
*h_I_*	Interface thickness	${\bar{\sigma }}_{f0}$	Initial strength of fibre
*δ*	Oxidation product thickness	*B*	Parabolic rate constant
*D_SiO_*_2_	Oxygen diffusion coefficient in SiO_2_	*V_r_*	Volume ratio of oxidation product to reactant
*V_SiO_*_2_	Volume of SiO_2_	*V_SiC_*	Volume of SiC
*M_SiO_*_2_	Molar mass of SiO_2_	*M_SiC_*	Molar mass of SiC
*ρ_SiO_*_2_	Density of SiO_2_	*ρ_SiC_*	Density of SiC
*e*_0_	Crack width of the matrix at room temperature	*T*_0_	Material preparation temperature
*σ*	External load	*α* *_m_*	Coefficient of thermal expansion of matrix
*α* *_f_*	Coefficient of thermal expansion of fibre	*E_f_*	Elastic modulus of fibre
*V_m_*	Volume fraction of the matrix	*N_O_*_2_	Gradient of oxygen molar flux
*R_O_*_2_	Consumption rate of oxygen in chemical reactions	*χ* *_O_*_2_	Molar fraction of oxygen
*C_c_*	Initial oxygen concentration	*S_SiO_*_2_	Surface area of SiO_2_ formed by oxidation
*V_f_*	Volume fraction of fibre	*V_c_*	Volume of composite material test segment
*L*_0_	Length of the test segment	*W*_0_	Width of the test segment
*H*_0_	Height of the test segment	*A_i_*	Existence of i-th level defect in fibre
*A_i_^j^*	The i-th level defect in the j-th microelement	*B_i_*	Event of fibre strength being σ_i_
*P*(*B_i_*)	Probability of event B_i_ occurring	*P*(*A_i_*)	Probability of event A_i_ occurring
*n_s_*	Random number	*P_i_*	Probability of i-th level defect in microelement
*σ* *_i_*	Strength of the defect	*i_in_*	Initial defect level of microelement
*i_oxi_*	Defect level after microelement oxidation	*τ* *_i_*	Shear stress of the interface phase
*τ_ult_*	Critical shear stress of the interface phase	*λ*	Oxidation influence constant
*α*_0_	Initial value of oxidation degree	*σ* *_f_*_0_	Normal stress in the fibre bonding zone
*α_c_*	Coefficient of thermal expansion of composite material	*E_c_*	Elastic modulus of composite material
*E_m_*	Elastic modulus of the matrix	*d_f_*	Sliding distance
*L*	Average spacing of matrix cracks	*U_m_*	Strain energy of the matrix
*U_cr_*	Critical strain energy value	*σ* *_m_*_0_	Normal stress in the matrix bonding zone
*L_ef_*	Length of the “influence zone”	*N*	Total number of fibre filaments
